# Thioredoxin-2 Modulates Neuronal Programmed Cell Death in the Embryonic Chick Spinal Cord in Basal and Target-Deprived Conditions

**DOI:** 10.1371/journal.pone.0142280

**Published:** 2015-11-05

**Authors:** Marc Pirson, Stéphanie Debrulle, André Clippe, Frédéric Clotman, Bernard Knoops

**Affiliations:** 1 Group of Animal Molecular and Cellular Biology, Institut des Sciences de la Vie (ISV), Université catholique de Louvain, 1348 Louvain-la-Neuve, Belgium; 2 Group of Neural Differentiation, Institute of Neuroscience (IONS), Université catholique de Louvain, 1200 Brussels, Belgium; Indian Institute of Integrative Medicine, INDIA

## Abstract

Thioredoxin-2 (Trx2) is a mitochondrial protein using a dithiol active site to reduce protein disulfides. In addition to the cytoprotective function of this enzyme, several studies have highlighted the implication of Trx2 in cellular signaling events. In particular, growing evidence points to such roles of redox enzymes in developmental processes taking place in the central nervous system. Here, we investigate the potential implication of Trx2 in embryonic development of chick spinal cord. To this end, we first studied the distribution of the enzyme in this tissue and report strong expression of Trx2 in chick embryo post-mitotic neurons at E4.5 and in motor neurons at E6.5. Using *in ovo* electroporation, we go on to highlight a cytoprotective effect of Trx2 on the programmed cell death (PCD) of neurons during spinal cord development and in a novel cultured spinal cord explant model. These findings suggest an implication of Trx2 in the modulation of developmental PCD of neurons during embryonic development of the spinal cord, possibly through redox regulation mechanisms.

## Introduction

Reactive oxygen (ROS) and nitrogen species (RNS) are molecules generated by the cell in pathophysiological situations but also as natural byproducts of their metabolism. These molecules can oxidize different cell components such as proteins, lipids or DNA, causing oxidative damage which can lead to cell death. To maintain ROS/RNS at non-toxic levels, where they may play a functional role such as in redox signaling, cells deploy a wide array of antioxidant enzymes [1]. Amongst these, thioredoxins (Trxs) appear to be key players in cytoprotection against oxidative insult but also in the redox regulation of many biological pathways [2]. Trxs use two reactive cysteine residues located in a conserved WCGPC motive to accomplish their reduction cycles. These ubiquitous enzymes act as disulfide bond reductants and, notably, serve as the main reductant for ROS/RNS scavengers peroxiredoxins (Prdxs). Vertebrates express two isoforms of Trxs, Trx1 and Trx2 [2]. Trx1 is localized in the cytosol, but is also found in the nucleus and secreted under certain conditions. Trx2, on the other hand, is exclusively mitochondrial [2–4]. Beyond their role in antioxidant cytoprotection, Trxs have also been shown to serve functions in redox regulation of several cellular processes through their ability to reduce disulfide bonds in many proteins including transcription factors and proteins implicated in cell signaling [5]. For instance, both Trx1 and Trx2 take part in the redox regulation of c-Jun N-terminal kinase (JNK) and p38 MAPK pathways, implicated in differentiation and programmed cell death (PCD), notably through their interaction with Ask-1 [6, 7]. In basal conditions, Ask-1 is inhibited by Trx1 and Trx2 in the cytosol and mitochondria, respectively. Oxidation of these Trxs results in the release of Ask-1 and its subsequent auto-activation leading to promotion of PCD via JNK-dependent signaling in the cytosol and cytochrome C release from the mitochondria.

Embryonic development entails the complex interaction of fundamental cellular processes such as proliferation, differentiation, migration and PCD. In addition to considerable circumstantial evidence, studies have also directly linked ROS/RNS and antioxidant systems, such as the Trx- or glutathione-dependent systems, to these developmental processes, notably in the central nervous system [5, 8–12]. For instance, proliferation as well as exit from cell cycle and differentiation of neural progenitors have been shown to be redox-controlled [13–15]. Moreover, neurite outgrowth, one of the hallmarks of neuronal differentiation, has also been reported to be modulated by redox-dependent processes [16–18]. Furthermore, oxidative stress has also been shown to play an essential role in naturally occurring developmental motor neuron PCD [19]. Conversely, antioxidant molecules have been implicated in the rescue of neurons from this ROS/RNS-induced PCD. For example, glutaredoxin-2 was shown to improve neuronal survival in zebrafish central nervous system (CNS) during development [17]. Similarly, developmental motor neuron death, reproduced in an explant culture system, was prevented by EUK-134, a catalase—superoxide dismutase mimetic [19].

In previous studies completed in our laboratory, we showed that Prdxs and Trxs are highly expressed in spinal cord motor neurons during embryonic development in the mouse [20]. Notably, Trx2 expression was particularly high in motor neurons at developmental stages coinciding with the onset of developmental PCD of motor neurons.

In the present study, we show that Trx2 is highly expressed in post-mitotic neurons at E4.5 and in motor neurons at E6.5 during chick embryonic spinal cord development. Using *in ovo* electroporation technique to overexpress or downregulate Trx2 during development, we go on to show that Trx2 significantly modulates neuron PCD *in vivo* as well as using an original approach via dissected spinal cord explant cultures.

## Materials and Methods

### Animal experimentation

Experimental procedures on animals were approved by the animal ethics committee of the Université catholique de Louvain and are in agreement with the European directive 2010/63/UE.

### Cloning of Gallus gallus Trx2 cDNA

cDNA was retrotranscribed from total RNA obtained from chicken heart, liver, lung and skeletal muscle. Tissues were obtained from an adult male chicken, kindly provided by a farm in Tilly (Belgium), after carbon dioxide euthanasia. Samples were dissected, homogenized in Trizol (Thermo Fischer Scientific) and retrotranscribed using Quantitect reverse transcription kit (QIAGEN) according to manufacturer’s instructions. Primers for polymerase chain reaction (PCR) amplification of chicken Trx2 were designed from NCBI reference sequence (NM_001031410.1). Forward primer was 5’-GGTTGTGCAGGGGTCACCTC-3’ and reverse primer was 5’-GAAGGAAGGGTTACAACATCG-3’. PCR was carried out with high fidelity FastStart Taq DNA polymerase (Roche). Surprisingly, amplification produced a 642-bp in place of the 482-bp sequence expected from *Gallus gallus* genome annotations. This size difference is detailed and discussed in Results and Discussion sections of this work. The amplicon was subcloned into PCR2.1 plasmid (Invitrogen) and sequenced. For subcloning, chicken Trx2 cDNA was amplified with forward primer 5’-CCTCGAATTCGGGAAGATGGCCCAGAGGCTGG-3’ and reverse primer 5’-CCACGCGGCCGCTTCAGGCTCCAATGAGTTTCTT-3’ containing restriction sites for EcoRI and Not1, respectively (restriction sites are underlined). After restriction, the sequence was ligated into pCMS-EGFP vector (Clontech) in which the *Gallus gallus* Trx2 coding sequence was under the control of a CMV promoter. eGFP is encoded by a separate gene under the control of a SV40 promoter.

### In ovo electroporation

Fertilized eggs were obtained from Wyverkens farm (Halles, Belgium) and put in a humidified incubator at 38°C. *In ovo* electroporation was performed at E2.5 or Hamilton-Hamburger stage (HH) 12–14. pCMS-EGFP vector, pCMS-EGFP vector with chick Trx2 cDNA sequence (Trx2 vector), standard control 3’-carboxyfluorescein (FITC) morpholino provided by Gene Tools (MoCTRL) or 3’-carboxyFITC morpholino targeted to chick Trx2 (MoTrx2) were injected into the neural tube at 2 μg/μl for DNA constructs and at 3 mM for morpholinos using a mouthpipet and microcapillaries. Thereafter, embryos were submitted to five 25 V square wave pulses with a TSS20 ovodyne electroporator and EP21 current amplifier. MoTrx2 (5’- TCAGTGCCAGCCTCTGGGCCATCTT -3’) was designed to cover 5’ region of Trx2 mRNA including the AUG. Forty-eight hours or 4 days after electroporation, embryos were removed from the egg and processed for corresponding analyses. At least 10 crysoections per embryo originating from at least 4 different embryos, though generally more, were used for the analyses performed in this work. Only embryos displaying strong eGFP or FITC signal were considered for subsequent processing. It is also of note that though electroporation clearly favors incorporation of the morpholinos on the ipsilateral side, a varying amount could also be detected on the contralateral side. For this reason, only slices showing a marked difference of morpholino signal on the ipsilateral side compared to the contralateral side were considered for the subsequent analyses.

### Western blotting

Forty-eight hours after electroporation, embryos were removed from the egg and decapitated. Spinal cords were dissected in PBS and the ipsi- and contraletral sides were separated and rapidly placed in liquid nitrogen. Frozen sample were homogenized in a buffer containing 150 mM NaCl, 1 mM EDTA, 1% Triton X-100, 10 mM Tris, pH 7.5, and protease inhibitor cocktail (Roche). Homogenates were centrifuged twice at 200 G to pellet debris, supernatants were recovered and stored at −20°C until use. Proteins were loaded on a 15% acrylamide sodium dodecyl sulfate-polyacrylamide gel electrophoresis (SDS-PAGE) in presence of DTT. After transfer, the nitrocellulose membranes were blocked in 5% nonfat dry milk in 150 mM NaCl, 20 mM Tris, pH 7.5 with 0.1% Tween (TTBS) at room temperature for 1 hour and then incubated with primary antibody diluted in TTBS overnight (see Table 1 for antibody information and dilutions). Blots were washed in TTBS, followed by incubation with horseradish peroxidase (HRP)-conjugated secondary antibody (Dako) for 1 hour. Blots were developed with the Western Lightning Chemiluminescence kit (Perkin Elmer) according to the manufacturer's instructions.

**Table 1:** Primary antibodies used in this study.

Antigen	Source	Dilution
Trx2	Rabbit polyclonal, obtained in Hormonology laboratory of Marloie (Belgium), No. UC177	1:500 in immunofluorescence 1:4000 in Western blotting
β-actin	Mouse monoclonal (AC-15), Sigma-Aldrich, catalog No. A1978	1:4000 in Western blotting
ATPB	Mouse monoclonal (3D5), Abcam, catalog No. ab14730	1:500 in immunofluorescence
NeuN	Mouse monoclonal (A60), Millipore, catalog No. MAB377	1:250 in immunofluorescence
Isl1/2	Mouse monoclonal (39.4D5), DSHB	1:5000 in immunofluorescence
Lhx3	Mouse monoclonal (67.4E12), DSHB	1:1000 in immunofluorescence
Lhx1/5	Mouse monoclonal (4F2), DSHB	1:2000 in immunofluorescence
Nkx2.2	Mouse monoclonal (74.5A5), DSHB	1:1000 in immunofluorescence
Evx1	Mouse monoclonal (99.1-3A2), DSHB	1:10 in immunofluorescence
Cleaved Casp-3 (Asp175)	Rabbit polyclonal, Bioke, catalog No. 9661S	1:200 in immunofluorescence

### Tissue processing

Forty-eight hours or 4 days after electroporation, embryos were removed from the egg and decapitated before fixation by immersion in 4% paraformaldehyde diluted in PBS (PFA 4%, pH 7.4) for 30 or 75 minutes depending on the developmental stage and cryopreservation in 30% sucrose diluted in PBS for 2-3h or overnight depending on the developmental stage. Tissues were embedded in cryomatrix on dry ice. The embedded samples were then kept at -80°C until processing. Cryosectioning was performed using a Leica CM3050 cryostat microtome. Samples were sectioned into 14 μm transversal sections, and sections were placed upon superfrost plus microscope slides (Thermo Scientific). Slides were stored at −80°C until immunolabeling experiments or TUNEL staining.

### Dissected spinal cord explant preparation and culture

Forty-eight hours after electroporation spinal cords from E4.5 chick embryos were dissected in PBS, opened dorsally and as much of the meninges and surrounding tissues as possible were removed. Open-book spinal cords were placed in liquid matrigel matrix (Corning) and allowed to set at 37°C for 30–45 min. The open-books were then cultured in DMEM supplemented with 15% fetal bovine serum and penicillin/streptomycin for 12h in a humidified incubator at 37°C in 5% CO_2_/95%-air. Then, spinal cords were fixed for 30 min in PFA 4% and cryopreserved in 30% sucrose diluted in PBS for several hours before embedding in cryomatrix on dry ice. The embedded samples were then kept at -80°C until processing. Cryosectioning and conservation was performed as described above.

### Antibodies and immunofluorescence assay

**Fig 1:**
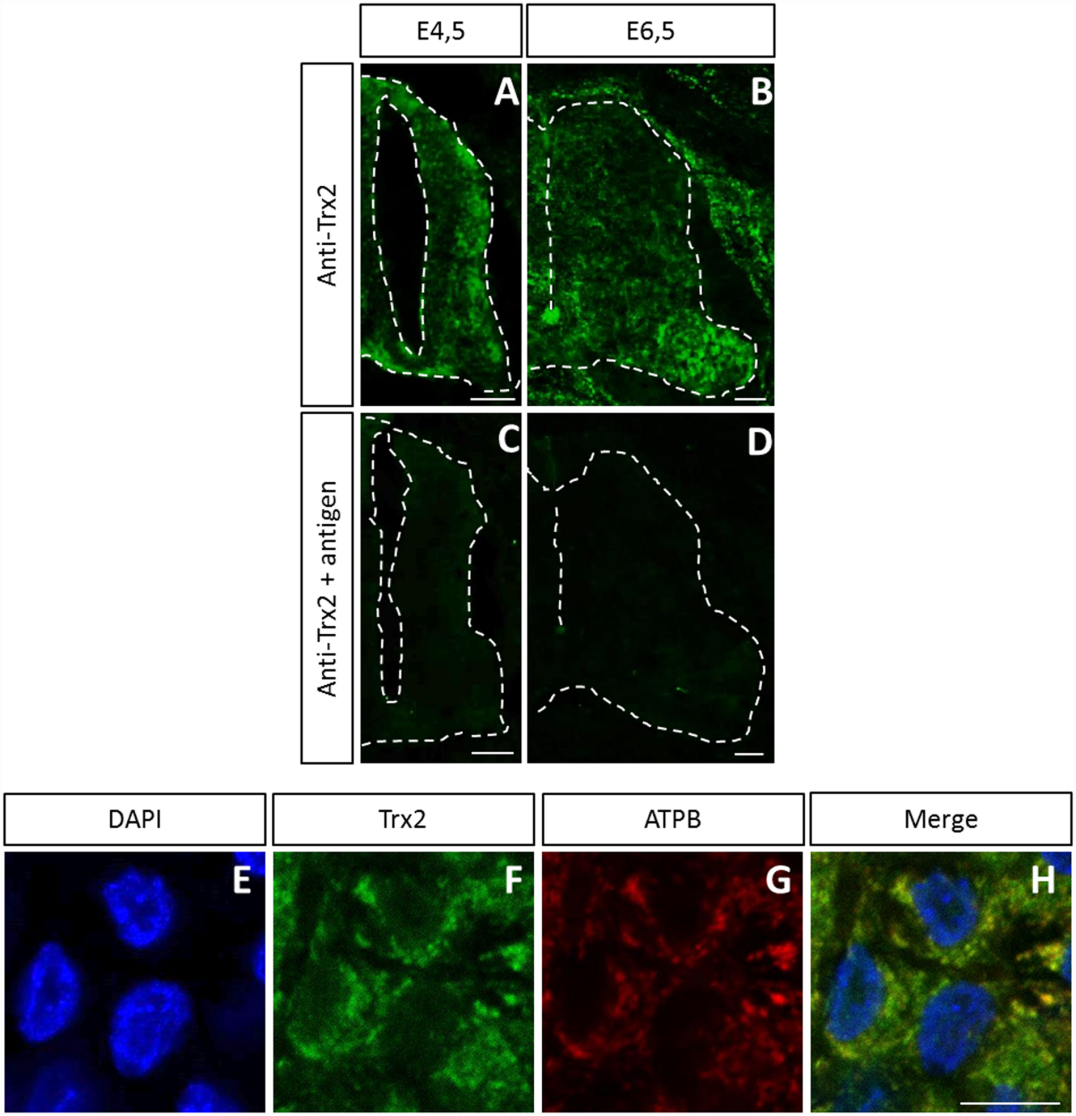
Trx2 antibody validation in chick spinal cord. (A-D) Immunofluorescence assay of E4.5 and E6.5 chick embryo spinal cords with anti-Trx2 (A, B) and anti-Trx2 preadsorbed with recombinant Trx2 (C, D). Colocalization of anti-Trx2 with mitochondrial marker ATPB (E-H). Scale bar = 50 μm (A-D) and 10 μm (E-H).

Polyclonal rabbit antibody directed to human Trx2 used in this work was previously validated in the embryonic mouse spinal cord [20]. Validation of this antibody in chick CNS was confirmed through the reduction of signal observed in Western blotting (data not shown) and immunofluorescence after preadsorption with recombinant human Trx2 (Fig 1A–1D). Moreover, signal associated to Trx2 overlaps with mitochondrial marker ATPB and overexpression of chicken Trx2 was also clearly detected with the antibody. Information and dilution for antibodies used in this work are reported in Table 1. Sections were washed in 0.1 M TBS with 0.25% Triton (TBS-T) before being blocked for 1 hour in 10% milk diluted in TBS-T. The slides were then incubated overnight with the antibodies diluted in TBS-T. The sections were then washed and incubated 1 hour with Alexa Fluor conjugated secondary antibodies in a dark moist chamber. Finally, samples were washed, incubated with 10 μg/ml DAPI (4′-6-diamidino-2-phenylindole; Roche) diluted in 50 mM Tris-base buffer (TB-DAPI) and mounted in Dako fluorescent mounting medium. Immunofluorescence images of cryosections were acquired on a Zeiss LSM 710 confocal microscope. All secondary antibodies gave signal in the spinal cord only in the presence of corresponding primary antibodies, but not when applied on spinal cord sections alone.

### TUNEL assay

For detection of DNA fragmentation, embryos were removed from the egg 2 or 4 days after electroporation and processed as described above. After cryosectioning, sections were rinsed with warm PBS (45–50°C) three times. The sections were then incubated with boiling 10mM pH 6 citric acid and microwaved for 5 min. Samples were then cooled by adding room-temperature distilled water. Following this, slices were incubated in 0.1% sodium citrate and 0.1% Triton X-100 for 5 min on ice and then in the terminal deoxynucleotidyl transferase-mediated dUTP nick end-labeling cocktail, containing tetramethylrhodamine (TMR)-dUTP, for 1 hour at 37°C according to the manufacturer's instructions (in situ cell death detection kit—TMR red; Roche). Finally, the sections were washed first in PBS, then in TB-DAPI before mounting as described above. Images were acquired on a Zeiss LSM 710 confocal microscope. TUNEL-positive cells were counted manually for E6.5 embryos and using ImageJ software for E4.5 open-book spinal cord preparations. The number of TUNEL-positive cells on the ipsilateral and contralateral sides of several slices were counted and compared for several individual embryos for test and control conditions.

### Statistical analysis

All statistical analyses were performed with SAS 9.4. software. Datasets were normalized using square root transformation for TUNEL counts in basal conditions and logarithm transformation for ratios (ipsi/contra) of TUNEL counts in explants cultures. Statistical comparison of differences between groups was performed using a mixed model on the normalized data sets. For both analyses, a probability value of p < 0.05 was considered statistically significant. Values are expressed as means ± confidence intervals.

## Results

### Gallus gallus Trx2 mRNA

**Fig 2:**
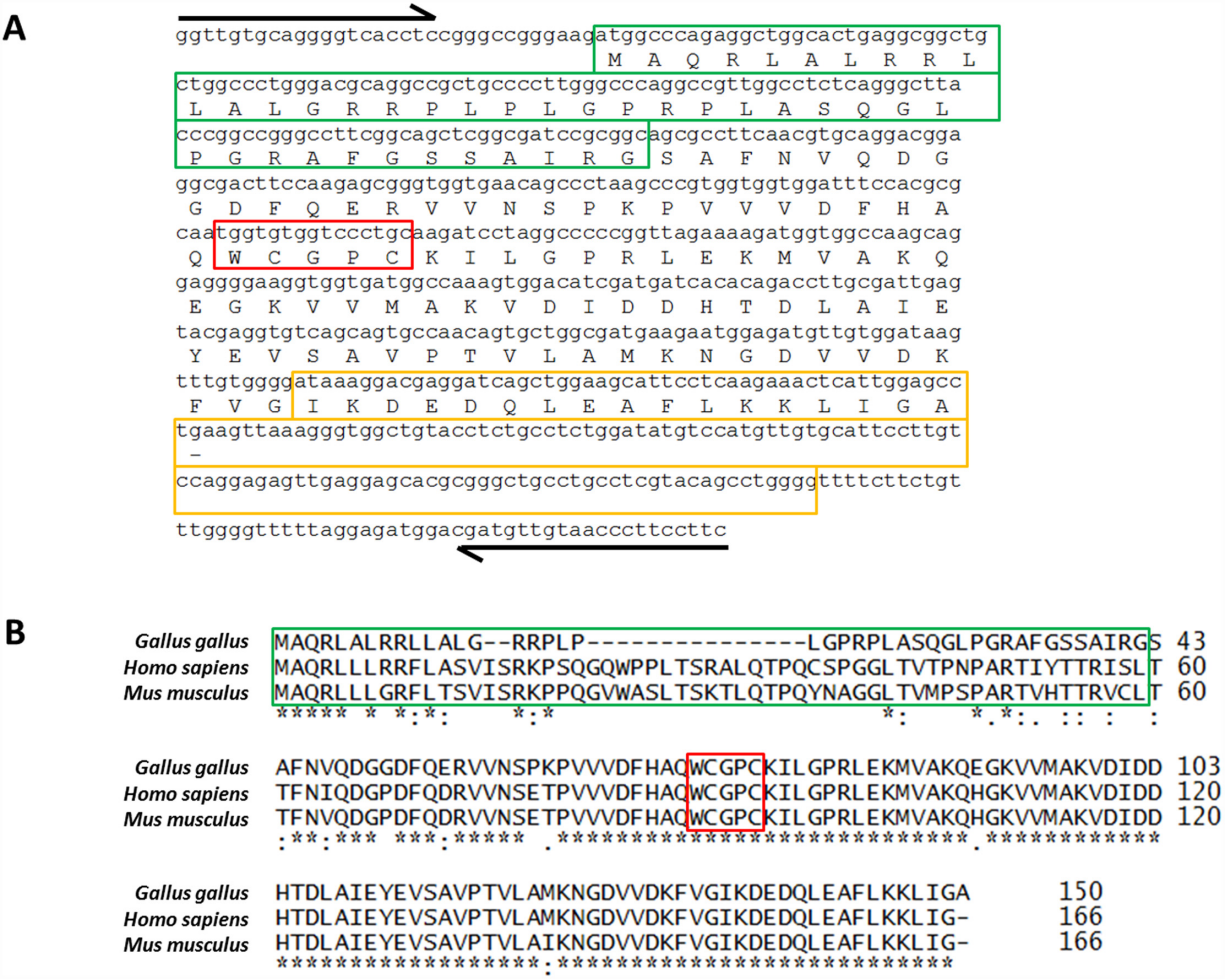
*Gallus gallus* Trx2 cDNA and amino acid sequence. (A) Sequence of *Gallus gallus* Trx2 PCR-amplified from total chick heart, liver, lung and muscle cDNA and predicted amino acid sequence. Amplification yielded a 642-bp amplicon instead of the 482-bp amplicon expected from the NCBI reference sequence (Accession number: NM_001031410.1). This difference is associated to a 160-bp sequence (indicated in yellow) located in the 3’-half of the whole *Gallus gallus* Trx2 cDNA. (B) Alignment of *Gallus gallus* Trx2 protein predicted from cDNA sequence with human (Accession number: Q99757) and mouse (Accession number: P97493) Trx2 amino acid sequences. Nucleotide sequences used to design forward and reverse primers for amplification are indicated by the black arrows. Mitochondrial targeting sequences (MTS) are indicated in green and conserved catalytic WCGPC motives are indicated in red.

In order to clone the coding sequence and design the morpholinos, *Gallus gallus* Trx2 cDNA was amplified and sequenced. Primers for Trx2 cDNA amplification were designed from the NCBI reference sequence for chick Trx2 cDNA (NM_001031410.1). Amplification of Trx2 from chick heart, liver, lung and muscle cDNA yielded a 642-bp sequence in place of the 482-pb sequence expected from the reference sequence (NCBI accession number NM_001031410.1 and Fig 2A). Alignment of the 642-bp sequence with the reference sequence suggested that this difference in electrophoretic mobility was due to an additional 160-bp sequence (indicated in yellow in Fig 2A) located in the 3’-half of the whole *Gallus gallus* Trx2 cDNA. Alignment of protein sequence predicted from the 642-bp chick Trx2 cDNA amplified in this work with human and mouse Trx2 NCBI reference sequences, accession numbers Q99757 and P97493, respectively, revealed important identity between amino acid sequences, in particular when omitting the predicted mitochondrial targeting sequence (indicated in green in Fig 2) which displays a lower degree of conservation than the enzyme *per se* (Fig 2B).

### Trx2 antibody validation

The Trx2 antibody used in this work was previously validated in the embryonic mouse spinal cord [20]. In order to validate the antibody in chick embryo spinal cord, immunofluorescence assays using the antibody or the antibody preadsorbed with the antigen were also performed on E4.5 and E6.5 chick embryos (Fig 1A–1D). Preadsorption of the antibody with the recombinant protein caused an important decrease in immunostaining. Trx2 antibody specificity was further supported through colocalization analysis with mitochondrial marker ATPB (see Fig 1E–1H).

### Trx2 is strongly expressed in NeuN-positive cells at E4.5 and in Isl1/2-positive cells at E6.5 in the embryonic chick spinal cord

**Fig 3:**
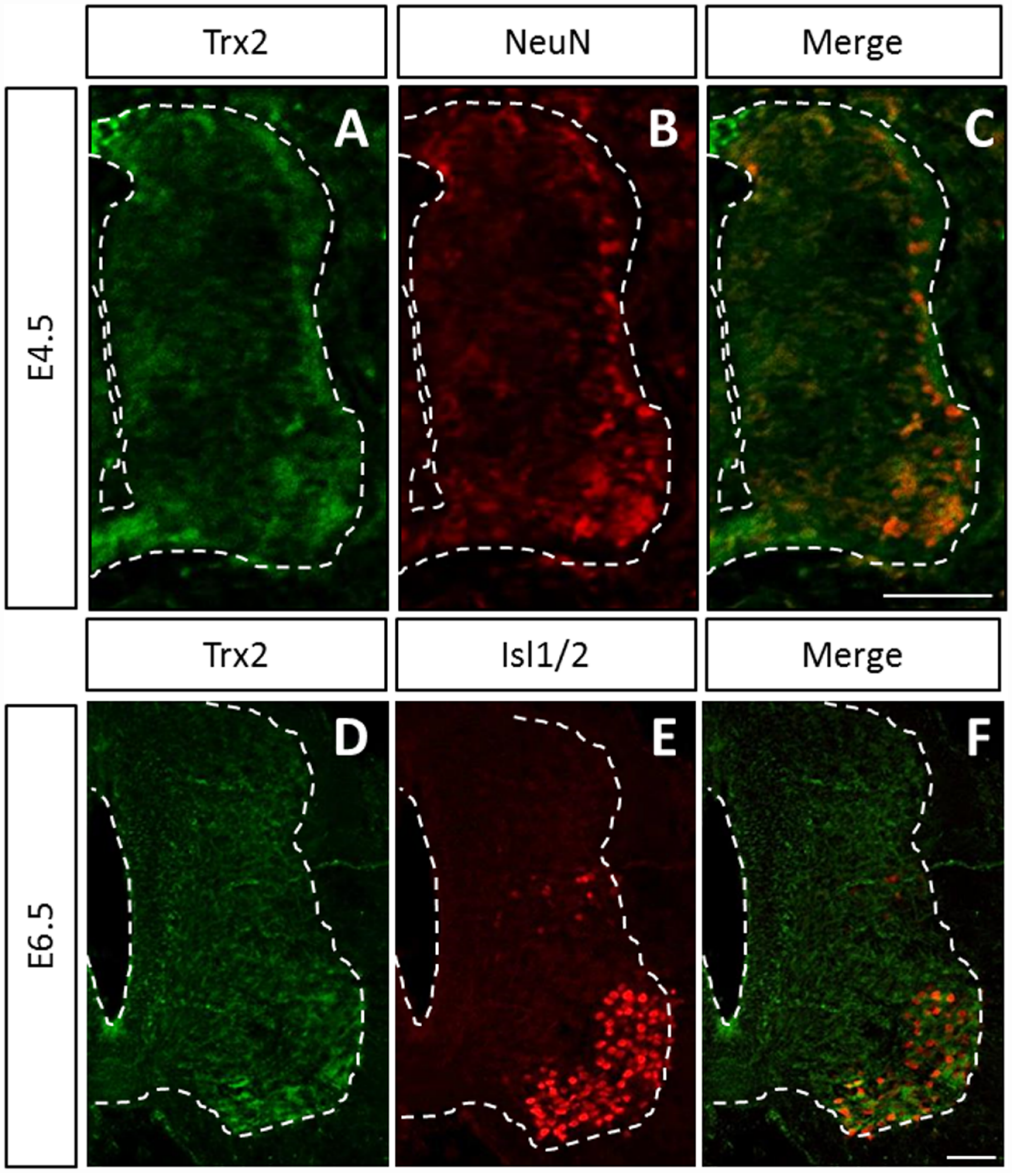
Trx2 colocalizes with NeuN-positive cells at E4.5 and Isl1/2 positive cells at E6.5. Colocalization of Trx2 with NeuN at E4.5 (A-C) and with Isl1/2 at E6.5 (D-F) in the embryonic chick spinal cord. Scale bar = 50 μm.

To uncover putative expression patterns of Trx2 during chick spinal cord development, immunofluorescence assays using Trx2 antibody were carried out at E4.5 and E6.5 (Fig 3). Anti-Trx2 antibody produced signal in all of the spinal cord but more prominently in lateral territories of the E4.5 spinal cord and in ventro-lateral territories at E6.5 (Fig 3A and 3D, respectively). This lateral staining colocalized with post-mitotic neuron marker NeuN at E4.5 (Fig 3A–3C) and with motor neuron marker Isl1/2 at E6.5 (Fig 3D–3F). It is of note that at both stages, Trx2 labeling was not exclusive to these cell populations. Indeed, Trx2 immunoreactivity was also visible in certain Isl1/2-negative cells located in the ventral spinal cord. Moreover, though Trx2 antibody showed intense staining in certain Isl1/2-positive cells, other Isl1/2-positive cells displayed more moderate immunoreactivity.

### Trx2 overexpression and downregulation

**Fig 4:**
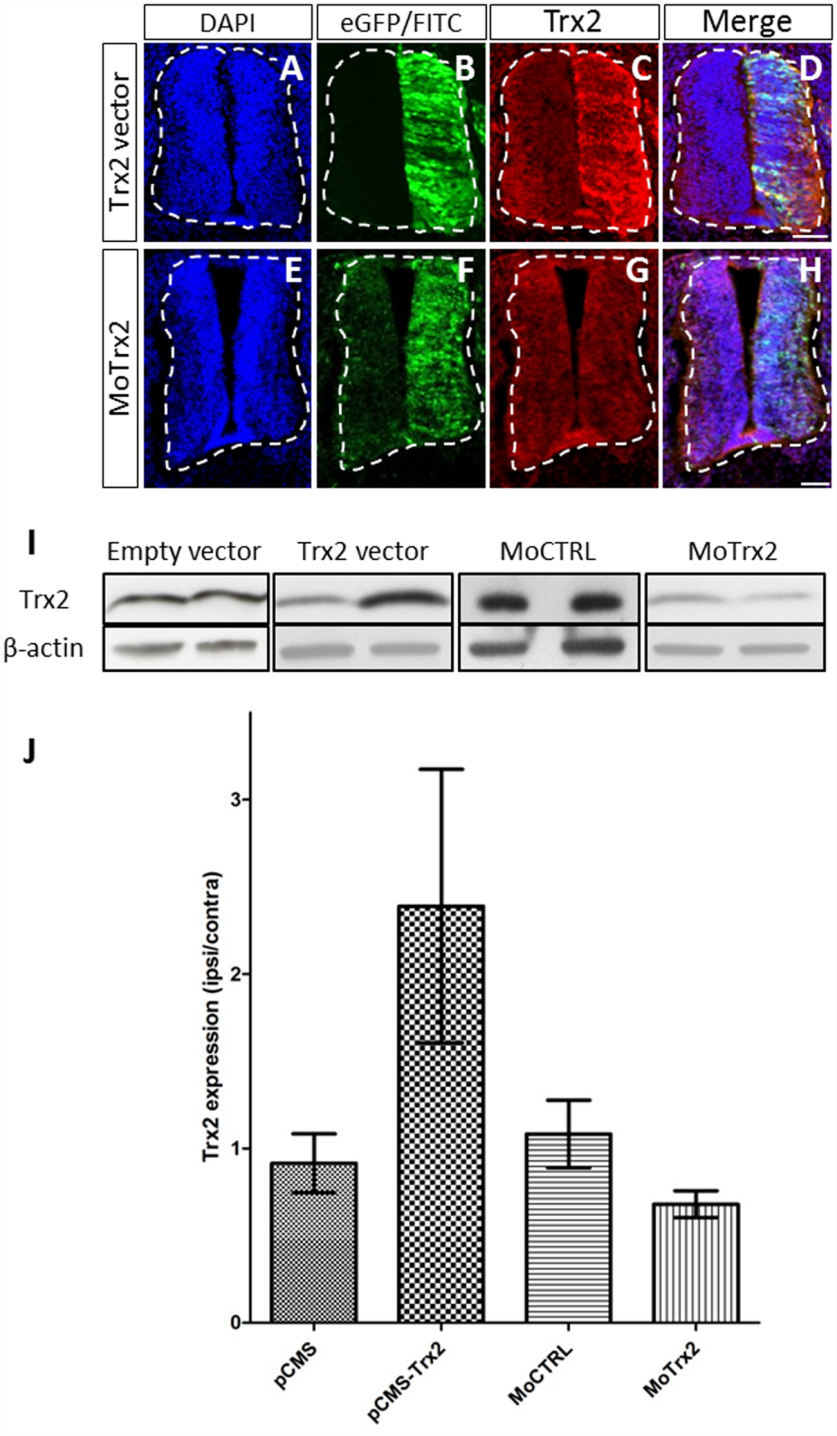
Trx2 overexpression and downregulation by *in ovo* electroporation. (A-H) Immunofluorescence detection of Trx2 in E4.5 chick embryo spinal cords electroporated with Trx2 vector or MoTrx2. (I) Western blotting analysis of the contralateral (non-electroporated; left well) and ipsilateral (electroporated; right well) sides of dissected spinal cords of E4.5 chick embryos electroporated with empty vector, Trx2 vector, MoCTRL or MoTrx2. (J) Densitometric analysis of Trx2 expression, shown as the ratio of the ipsilateral measurement on the contralateral measurement. Scale bar = 50 μm.

In order to study the effect of Trx2 during chick spinal cord development, a Trx2 expressing vector (Trx2 vector) or a morpholino targeting Trx2 mRNAs (MoTrx2) was electroporated into the neural tube of chick embryos. Negative controls were also performed using the empty vector (empty) and a control morpholino (MoCTRL). Immunofluorescence analyses of embryos electroporated with the Trx2 vector show an effective overexpression of both eGFP (Fig 4B), serving as a control of electroporation, and Trx2 (Fig 4C). Overexpression of Trx2 is further confirmed through Western blotting on homogenates of ipsilateral (electroporated) or contralateral (non-electroporated) parts of dissected E4.5 chick embryo spinal cords (Fig 4I). Though immunofluorescence assays enabled us to easily detect overexpression of the protein, the background produced with the anti-Trx2 antibody as well as loss of FITC staining during the immunofluorescence procedure made downregulation difficult to highlight in this way (Fig 4G). However, Western blotting analysis revealed that MoTrx2 induced a marked decrease in Trx2 staining on the ipsilateral side compared to the contralateral side (Fig 4I). Densitometry analysis further supported these results, Trx2 vector producing a 140% increase in Trx2 on the ipsilateral side compared to the contralateral side and MoTrx2 yielding a more modest 32% decrease on the ipsilateral side compared to the contralateral side (Fig 4J). Conversely, the electroporation of the empty vector (empty) or control morpholino (MoCTRL) did not lead to a shift in Trx2 band intensity.

### Overexpression or downregulation of Trx2 do not affect neuronal population markers at E4.5

**Fig 5:**
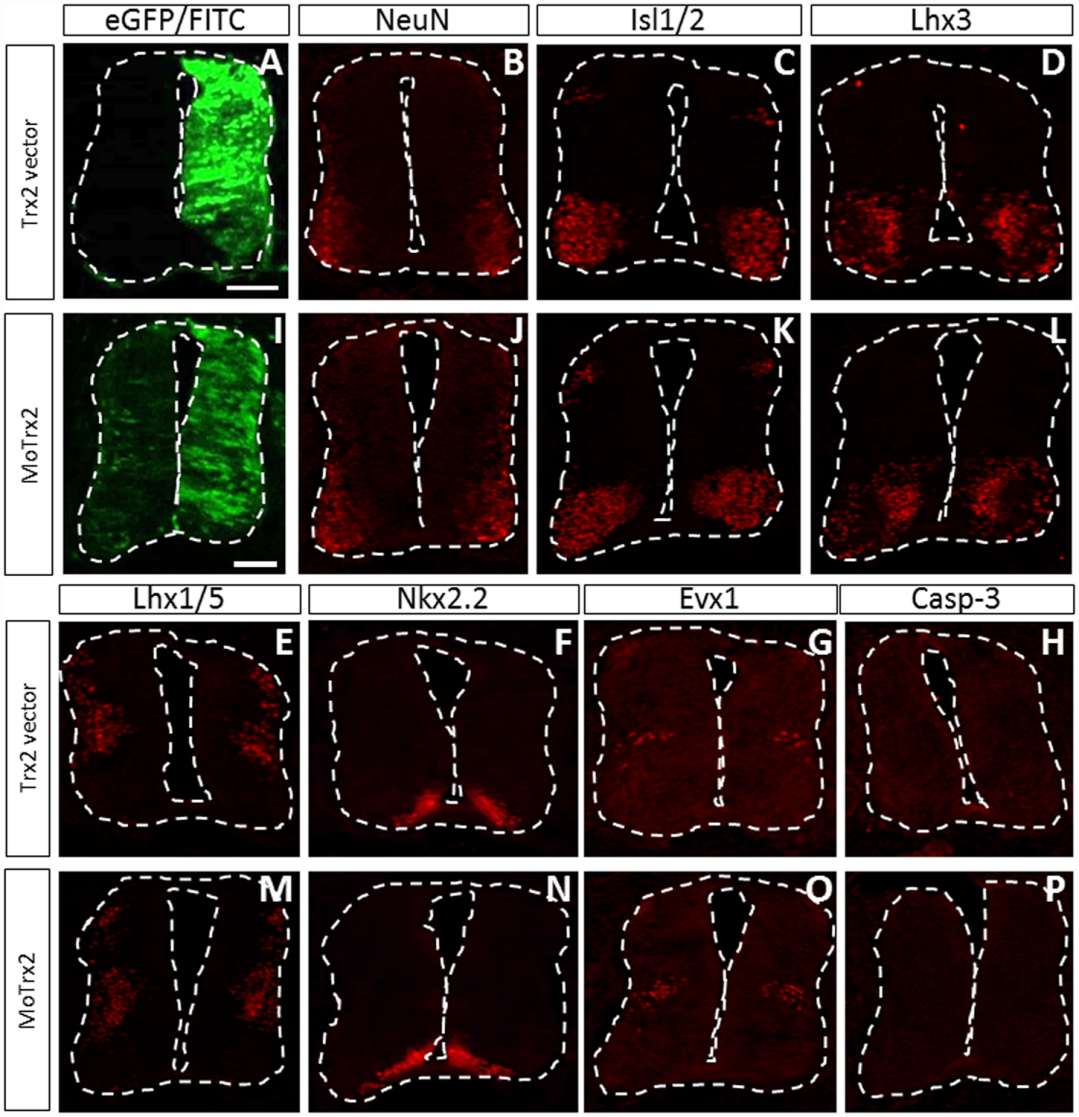
Overexpression or downregulation of Trx2 does not affect markers of neuronal populations in the spinal cord of E4.5 chick embryos. E4.5 chick embryos electroporated with Trx2 vector (A-H) and MoTrx2 (I-P) were probed with several markers of neuronal subpopulations (NeuN, Isl1/2, Lhx3, Lhx1/5, Nkx2.2, Evx1) and a marker of apoptosis (cleaved Casp-3). Scale bar = 50 μm.

Since Trx2 was strongly expressed in post-mitotic neurons at E4.5, a developmental stage at which neurogenesis and neuronal specification are actively taking place [21], we wanted to determine whether overexpression or downregulation of Trx2 could alter expression patterns of different neuronal population markers. To this end, immunofluorescence assays using several specific markers of neuronal populations were performed on spinal cord of E4.5 chick embryos electroporated with Trx2 vector or MoTrx2 (Fig 5). Immunodetection was performed to detect NeuN, a marker of post-mitotic neurons, motor neuron and DI3 interneuron marker Isl1/2, Lhx3 which is exclusively detected in V2 ventral interneurons and certain motor neurons, Lhx1/5 which is a marker of DI2, 4 and 6 dorsal interneurons as well as V0, V1 and V2b ventral interneurons and some motor neurons, Nkx2.2 which is present in V3 interneuron precursors and newly-born neurons and, finally, Evx1 which is detected in a part of V0 interneurons. No marked difference between the ipsilateral and contralateral sides was observed for these markers. In order to exclude minor toxic effects associated to the overexpression of Trx2 or to the morpholinos, electroporated spinal cords were also probed for early apoptosis marker cleaved caspase-3 (Casp-3). This antibody produced no significant staining in the spinal cord at this stage.

### Overexpression and downregulation of Trx2 mildly but significantly affect developmental PCD of neurons at E6.5

**Fig 6:**
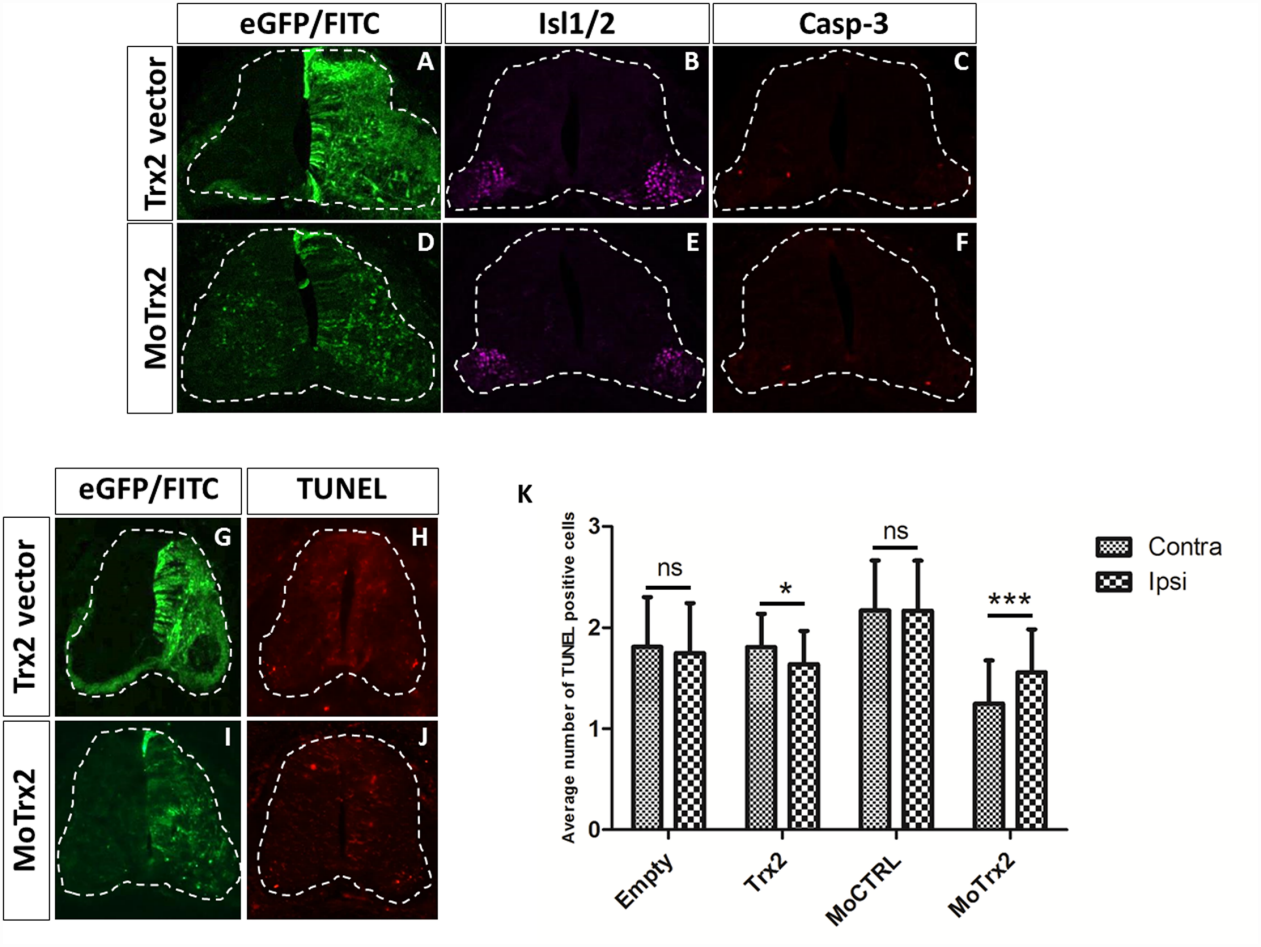
Trx2 overexpression and downregulation affect neuron PCD in the E6.5 chick spinal cord. (A-F) Immunofluorescence detection of motor neuronal marker Isl1/2 and apoptosis marker cleaved Casp-3 and (G-J) TUNEL staining in the spinal cord of E6.5 chick embryos electroporated with Trx2 vector or MoTrx2. (K) Comparison of the average TUNEL-positive cell count on the ipsi- and contralateral sides of spinal cord slices of E6.5 chick embryos electroporated with empty vector (N = 4), Trx2 vector (N = 9), MoCTRL (N = 4) or MoTrx2 (N = 5). Statistical significance is indicated as follows: ns = non-significant, * = p< 0.05, *** = p<0.0005.

Trx2 immunodetection showed marked staining in motor neurons at E6.5. At this developmental stage motor neurons are undergoing a period of naturally occurring PCD [22]. In order to examine the effect of overexpression or downregulation of Trx2 on this process, immunofluorescence assay using motor neuronal marker Isl1/2 and cell death marker Casp-3 was executed on spinal cord of E6.5 chick embryos electroporated with Trx2 vector or MoTrx2 (Fig 6A–6F). Qualitatively, signal for Isl1/2 and Casp-3 was not markedly different on the ipsilateral side with regards to the contralateral side. TUNEL technique was also used to highlight DNA fragmentation associated to advanced stages of caspase-dependent and caspase- independent cell death (Fig 6G–6J). Mild differences between the ipsi- and contralateral sides of E6.5 chick spinal cords electroporated with Trx2 vector or MoTrx2 were observed. Indeed, embryos electroporated with Trx2 vector presented less TUNEL-positive cells on the ipsilateral side than on the contralateral side while spinal cord electroporated with MoTrx2 showed the opposite tendency.

To confirm this trend, counts of TUNEL-positive cells on ipsi- and contralateral sides of spinal cords electroporated with empty vector, Trx2 vector, MoCTRL or MoTrx2 were carried out and these data were submitted to statistical analysis (Fig 6K). The results confirmed that the average number of TUNEL-positive cells on the ipsilateral side was significantly lower for spinal cord treated with Trx2 vector (p = 0.017) and significantly higher for spinal cords electroporated with MoTrx2 (p = 0.0003). Electroporation of empty vector and MoCTRL did not yield a significant difference in TUNEL counts on the ipsi- and contralateral sides (p = 0.1907 and 0.9820, respectively).

### Trx2 downregulation significantly affects survival of neurons in target-deprived spinal cord explants

Though our results reached statistical significance, the alteration of basal levels of developmental neuron PCD in the E6.5 chick embryo by Trx2 overexpression or downregulation remained modest. Technical issues such as the important variability associated to the low number of TUNEL-positive cells per slice or the important dilution the vector or morpholinos is subjected to after electroporation might impact our data and attenuate differences. In order to bypass these concerns, the effect of overexpression and downregulation of Trx2 was examined in dissected E4.5 spinal cords cultured for 12h. As shown by Sanchez-Carbente et al. (2005), dissection and organotypic culture of mouse spinal cords strongly increase motor neuron death as survival of these cells is tightly linked to the presence of target tissue [19, 23]. Here, we report a similar effect in chick embryonic spinal cord explant cultures. Indeed, compared to TUNEL staining *in vivo* at E6.5, E4.5 cultured explants showed a very strong increase in TUNEL-positive cells in the ventral part of the spinal cord (data not shown). Furthermore, TUNEL staining indicated that onset of this neuronal death took place between 6h and 12h of culture while after 24h of culture, close to all motor neurons had undergone cell death processes as revealed by a marked decrease Isl1/2 immunostaining (data not shown).

**Fig 7:**
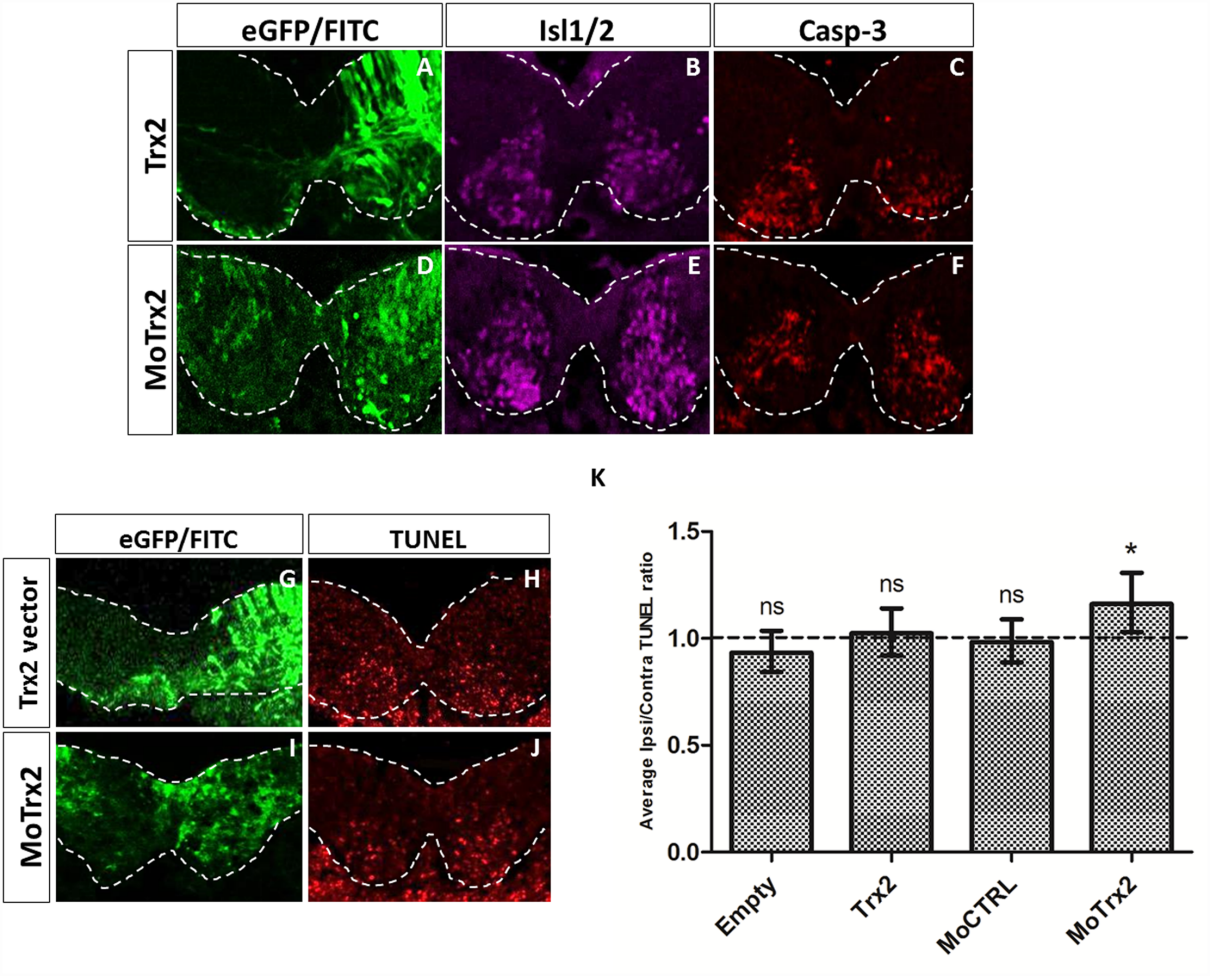
Trx2 downregulation increases neuron PCD in target-deprived spinal cord explants. (A-F) Immunofluorescence detection of motor neuronal marker Isl1/2 and apoptosis marker cleaved Casp-3 and (G-J) TUNEL staining in E4.5 chick spinal cords electroporated with Trx2 vector and MoTrx2 after culture for 12h, (K) Comparison of the average TUNEL-positive cell count of the ipsi- and contralateral sides of E4.5 chick spinal cord explants electroporated with empty vector (N = 6), Trx2 vector (N = 6), MoCTRL (N = 6) or MoTrx2 (N = 5). Statistical significance is indicated as follows: ns = non-significant, * = p< 0.05.

Immunofluorescence assay using motor neuronal marker Isl1/2 as well as apoptosis marker Casp-3 was carried out on E4.5 spinal cord explants cultured for 12h (Fig 7A–7F). Though spinal cords electroporated with Trx2 vector showed little to no changes in Isl1/2 immunostaining, Casp-3 signal appeared marginally elevated on the ipsilateral side compared to the contralateral side in embryos electroporated with the Trx2 vector and a more pronounced reduction was observed after MoTrx2 electroporation. DNA fragmentation assayed by TUNEL staining displayed similar trends to Casp-3 immunodetection (Fig 7G–7J), Trx2 vector yielding slightly less TUNEL-positive cells and MoTrx2 giving rise to an increased number of TUNEL-positive cells on the ipsilateral side compared to the contralateral side (Fig 7G–7J). Statistical analysis of the average ipsi/contralateral ratios of TUNEL-positive cell counts showed no significant effect for the empty vector, Trx2 vector or MoCTRL (p = 0.1776, 0.6489 and 0.7299 respectively) while MoTrx2 produced a significantly increased ratio of ipsi/contralateral TUNEL-positive cells (p = 0.0167).

## Discussion

In this study, we report the cloning of *Gallus gallus* Trx2 cDNA and considerable immunoreactivity associated to the enzyme in post-mitotic neurons at E4.5 as well as in motor neurons at E6.5 during chick embryonic spinal cord development. Thereafter, using *in ovo* electroporation, we assayed the effect of Trx2 gain and loss of function during chick spinal cord development. The main finding of this work is the statistically significant effect of Trx2 overexpression or silencing on the modulation of neuronal PCD *in vivo* and in target-deprived spinal cord explant cultures. In this section, we will go over these results and, taking advantage of current scientific literature, attempt to place them in their biological context.

### Trx2 cDNA and protein in Gallus gallus

Amplification of Trx2 cDNA from chick tissue cDNA yielded a 642-bp PCR product instead of the 482-bp amplicon expected from the NCBI reference sequence for *Gallus gallus* Trx2 mRNA (NM_001031410.1). Reference Trx2 mRNA sequence was produced by Caldwell et al. (2005) from a library of chicken bursal lymphocyte cDNAs [24]. Sequencing revealed that this difference corresponds to a 160-bp sequence, absent from the sequence published by Caldwell and colleagues, located in the 3’-half of the full Trx2 cDNA and containing an in-frame STOP codon. This 160-bp sequence is equally present in chicken genomic sequences. The protein sequence predicted from the Trx2 cDNA sequence cloned in the present study was similar to previously predicted chicken Trx2 protein and shows considerable identity with human and mouse Trx2 sequences, up to 71% when considering the full sequence and reaching 93% when omitting the mitochondrial targeting sequences which is expected to display a higher variability. Conversely, peptide sequence predicted from the mRNA described by Cadwell et al. (2005) [24] shows no homologues. These data suggest the existence of only one isoform of Trx2 in *Gallus gallus*, corresponding to the 642-bp amplicon obtained in this work, which encodes a 150 amino acid protein.

### Trx2 is highly expressed in post-mitotic neurons at E4.5 in the embryonic chick spinal cord

With aim of obtaining clues as to putative roles of Trx2 during development of the spinal cord, the distribution of the enzyme was analyzed in the chick embryo via immunofluorescence assays. Our results highlight an increased expression of Trx2 in post-mitotic neurons at E4.5. This might be linked to the intense metabolic activity of these cells. Indeed, post-mitotic neurons are exposed to oxidative stress and might therefore require increased activity of antioxidant enzymes such as Trx2 [25–27]. However, at this stage, neural progenitors are also actively proliferating, migrating laterally and differentiating into several types of neurons. It is therefore possible that Trx2 is implicated in these or other cellular processes taking place in post-mitotic neurons [21]. This is consistent with the implication of redox signaling and antioxidant enzymes in neurodifferentiation processes. Indeed, differentiation of cortical neural progenitors was shown to depend on subtle variations in intracellular redox state and suppression of ROS-dependent JNK signaling by Prdx1-2 was reported to play a pivotal role in maintaining the proliferative state of embryonic stem cells during neurogenesis [14, 28]. Yan et al. (2009) also showed that Prdx1 is an essential actor in differentiation of spinal cord motor neurons [15]. Prdx1 appears to exert this function through the reduction of a disulfide in GDE2 and downstream inactivation of Notch signaling [15, 29, 30]. Moreover, NGF-mediated neurodifferentiation of PC12 cells was demonstrated to depend on redox regulation by ROS as well as by Trx1 [31, 32]. However, this strong Trx2 expression might also be a consequence of differentiation.

### Trx2 is highly expressed in motor neurons at E6.5 in the embryonic chick spinal cord

Trx2 is also highly expressed in Isl1/2-positive motor neurons at E6.5. At this stage, chick spinal cord motor neurons are extending neuronal projections to connect their target tissue and undergoing a period of naturally occurring PCD [33]. Indeed, during spinal cord development motor neurons are generated in excess and an important fraction of these motor neurons undergoes a process of physiological PCD which has been shown to be controlled by target-dependent and independent survival or death cues [34]. This developmental motor neuron PCD has also been reported to be dependent on the presence of ROS [19]. In addition, both ROS and antioxidant enzymes such as Trx2 have been directly or indirectly linked to the onset and signaling cascades implicated in cell death pathways [35–37]. Coincidentally, Trx2 is also strongly expressed in mouse motor neurons at the developmental stages at which this motor neuron PCD takes places [20]. These data could therefore indicate an implication of Trx2 in the regulation of this process.

### Overexpression and downregulation of Trx2 significantly affect developmental PCD of spinal cord neurons

In order to study the putative functional role of Trx2 during chick spinal cord development, a gain and loss of function model was developed using *in ovo* electroporation of a Trx2 overexpression vector (Trx2 vector) and a morpholino directed to Trx2 mRNA (MoTrx2). Before initiating the functional study, confirmation of the overexpression and downregulation was established via immunofluorescence assay and Western blotting analysis. While these data confirm that Trx2 vector leads to an increase of Trx2 and MoTrx2 decreases enzyme expression, differences appear to be relatively mild. However, this analysis certainly underestimates the degree of the overexpression and downregulation. Indeed, upon electroporation, ipsilateral spinal cord homogenates will contain electroporated cells but also a varying amount non-electroporated cells that may dilute the effect. It is also of note that though electroporation clearly favors incorporation of the morpholinos on the ipsilateral side, a varying amount of morpholinos could also be detected on the contralateral side and could therefore reduce the relative ipsi/contra ratio of Trx2.

As mentioned previously, the high Trx2 expression in post-mitotic neurons in the E4.5 chick spinal cord could suggest the implication of Trx2 in processes such as proliferation, migration or differentiation of neurons [21]. In order to test this hypothesis, immunofluorescence assays using specific markers of different neuronal subpopulations were performed on E4.5 spinal cord slices of chick embryos electroporated with Trx2 vector or MoTrx2. When comparing the electroporated side to the contralateral side, no clear differences in size or morphology of the different neuronal subpopulations could be detected.

Trx2 also showed marked staining in Isl1/2-positive motor neurons at E6.5, an embryonic stage at which motor neuronal populations undergo target-dependent PCD [22]. To determine whether the intense expression of Trx2 in this cell population could be linked to this process, motor neuron populations and cell death were examined in E6.5 spinal cords of electroporated chick embryos. Motor neuronal populations were identified by immunofluorescence using motor neuron marker Isl1/2 while cell death was visualized by immunofluorescence using early apoptosis marker Casp-3 and via TUNEL assay. Differences in immunoreactivity of Isl1/2 and Casp-3 between the ipsi- and contralateral sides of embryos electroporated with Trx2 vector and MoTrx2 were relatively minimal. However, an interesting trend appeared after TUNEL staining. Spinal cord slices originating from embryos electroporated with Trx2 vector showed an increased number of TUNEL-positive cells on the ipsilateral side compared to the contralateral side. An opposite tendency was observed in spinal cords treated with MoTrx2. To establish the significance of these effects, counts of TUNEL-positive cells were performed on slices originating from E6.5 spinal cords of chick embryos electroporated with the empty vector, Trx2 vector, MoCTRL and MoTrx2. Data show a significant decrease in the average number of TUNEL-positive cells on the ipsilateral side with regards to the contralateral side in embryos electroporated with the Trx2 vector while MoTrx2 produces a significant increase. These results suggest that Trx2 is capable, to a certain extent, of modulating PCD of neurons at this stage. It is of note that though motor neurons are the main cell population undergoing this process during embryogenesis, PCD of interneurons has also been shown to take place to a certain degree at prenatal stages [38–40]. It is therefore important to remain cautious as to the strict association of TUNEL staining observed in this work to motor neuron PCD alone.

In order to further confirm these results and avoid bias associated to the small number of TUNEL-positive cells per slice, we optimized a new protocol for assaying the impact of Trx2 gain or loss of function in the developing chick spinal cord. Indeed, motor neuron PCD has been shown to depend on survival cues originating form target tissue [33, 34, 41]. Furthermore, culture of spinal cord tissue or cells in the absence of synaptic targets has been shown to induce an important increase in motor neuron death [19]. In order to amplify the neuronal PCD observed *in vivo*, E4.5 spinal cords of electroporated chick embryos were dissected and cultured for 12h. As expected, withdrawal of target tissue led to a marked increase in motor neuron death. This procedure also has the additional benefit that spinal cords are assayed two days after electroporation instead of four days thus increasing embryo survival rates and reducing dilution of the electroporated plasmid or morpholinos that takes place during division of progenitor cells. The adaptation of this technique in the chick embryo and its potential combination with *in ovo* electroporation offers a novel approach to study genes involved in neuronal survival and cell death in the context of development or pathophysiological situations.

Motor neuron populations and cell death were examined in this model by immunofluorescence with markers Isl1/2 and Casp-3 as well as with TUNEL staining. Interestingly, while downregulation of Trx2 led to a significant increase in TUNEL-positive cells, Trx2 overexpression failed to significantly reduce cell death. Firstly, these results confirm that reduction of Trx2 levels promotes PCD of neurons, particularly in absence of target tissue. The lack of effect observed for Trx2 overexpression in cultured spinal cord explants, as opposed to the significant effect observed for this treatment in basal conditions, could be an indicator that Trx2 interacts with other cellular components to mediate its cytoprotective effect. We hypothesize that at normal levels of neuron PCD, overexpression of Trx2 would provide sufficient cytoprotective stimulation to reduce the effect of the death signaling cascade. In culture conditions, however, death signaling would be much more consequent and though Trx2 is overexpressed, other elements of the cytoprotective system would be limiting. Conversely, downregulation of Trx2 would negatively impact survival of neurons both in basal and target-deprived conditions.

As mentioned previously, the naturally occurring elimination of motor neurons has been shown to be dependent on the presence of ROS and mitochondrial Trx2 is well known to protect cells from ROS-induced oxidative insult [7, 19, 42–44]. Our results could stem from this cytoprotective activity of Trx2. In basal conditions, Trx2 overexpression would provide neurons with an antioxidant boost, reducing protein disulfides resulting from the elevated concentrations of ROS in motor neurons and increasing turnover of mitochondrial ROS-scavenger Prdx3 [8]. On the contrary, downregulation would leave cells more vulnerable to oxidative damage both in basal conditions and in absence of target tissue. Several studies have linked Trx2 to cell survival and the control of cell death. First, knockout of Trx2 in mouse has proven to be embryonic lethal at E10.5 with embryos showing abnormal closing of the neural tube and massive increase of apoptosis [45]. Moreover, Trx2-deficient chicken DT40 cells exhibit clear hallmarks of cell death including increase in ROS, release of cytochrome C and activation of Casp-3 and Casp-9 [46]. Conversely, transfection of human Trx2 in the same cell line rescued cells from death through an active site-independent mechanism [47]. Modulation of PCD by Trx2 could also take place via its interactions with signaling molecules such as Ask-1 or p66Shc [7, 48, 49]. The variation in TUNEL-positive cells associated to Trx2 overexpression or downregulation, though significant, both *in vivo* and in explant culture, is still relatively mild. This could suggest that Trx2 is one of several factors that modulate survival or death of neurons during development. Therefore the effect of overexpression would be limited by the cellular components the enzyme interacts with. Indeed, regulation of cell survival and death is often complex and integrates several signaling inputs. Therefore, alteration of Trx2 levels might only impact the process to a certain degree. Conversely, the deleterious effect of downregulation might be attenuated by compensatory mechanisms provided by other protein disulfide oxidoreductases such as mitochondrial Grx2 for instance [50]. This hypothesis seems likely in light of growing evidence supporting the existence of a considerable crosstalk between the Trx/TrxR system and GSH-dependent systems [51–53]. In particular, strong upregulation of Grx2 has recently been reported in mitochondrial TrxR2^-/-^ mice embryonic fibroblasts [54]. As mentioned above, similarly to the Trx system, GSH-dependent systems have been reported to play major roles in developmental processes [8–10, 55].

## Conclusion

In this work, we report the cloning of a chick Trx2 cDNA and the identification of the sequence that corresponds to the *bona fide Gallus gallus* Trx2 mRNA as opposed to the current NCBI reference sequence. We also describe expression patterns of the Trx2 protein in the developing chick spinal cord, notably a high expression in motor neurons at a stage where these cells undergo developmental PCD. Furthermore, we show that Trx2 is capable of significantly modulating this process in basal conditions but also, by using a novel electroporated spinal cord explant culture technique, in the absence of survival cues originating from target tissue. These results highlight Trx2 as an actor of the complex regulation mechanisms controlling developmental PCD of spinal cord neurons and further elucidation of the molecular mechanisms involved may provide key insight into the redox regulation of this process.

## Supporting Information

S1 Supporting Information Data of TUNEL count of E6.5 chick embryo spinal cords after electroporation with Trx2 vector, MoTrx2 and corresponding controls. (XLSX)

S2 Supporting Information Data of TUNEL count of cultured E4.5 chick embryo spinal cord explants after electroporation with Trx2 vector, MoTrx2 and corresponding controls. (XLSX)

## Abbreviations

Casp-3: cleaved caspase-3
CNS: central nervous system
E: embryonic day
eGFP: enhanced green fluorescent protein
Grx: glutaredoxin
HH: Hamilton-Hamburger stage
PCD: programmed cell death
PFA: paraformaldehyde
Prdx: peroxiredoxin
RNS: reactive nitrogen species
ROS: reactive oxygen species
Trx: thioredoxin
TUNEL: terminal deoxynucleotidyl transferase-mediated dUTP nick end-labeling
